# Potential impact of *ADAM‐10* genetic variants with the clinical features of oral squamous cell carcinoma

**DOI:** 10.1111/jcmm.17728

**Published:** 2023-03-22

**Authors:** Yi‐Tzu Chen, Chiao‐Wen Lin, Ying‐Erh Chou, Shih‐Chi Su, Lun‐Ching Chang, Chia‐Yi Lee, Ming‐Ju Hsieh, Shun‐Fa Yang

**Affiliations:** ^1^ School of Dentistry Chung Shan Medical University Taichung Taiwan; ^2^ Department of Dentistry Chung Shan Medical University Hospital Taichung Taiwan; ^3^ Institute of Oral Sciences Chung Shan Medical University Taichung Taiwan; ^4^ School of Medicine Chung Shan Medical University Taichung Taiwan; ^5^ Department of Medical Research Chung Shan Medical University Hospital Taichung Taiwan; ^6^ Whole‐Genome Research Core Laboratory of Human Diseases, Chang Gung Memorial Hospital Keelung Taiwan; ^7^ Department of Dermatology Drug Hypersensitivity Clinical and Research Center, Chang Gung Memorial Hospital Linkou Taiwan; ^8^ Department of Mathematical Sciences Florida Atlantic University Boca Raton Florida USA; ^9^ Institute of Medicine Chung Shan Medical University Taichung Taiwan; ^10^ Nobel Eye Institute Taipei Taiwan; ^11^ Oral Cancer Research Center, Changhua Christian Hospital Changhua Taiwan; ^12^ Department of Post‐Baccalaureate Medicine, College of Medicine National Chung Hsing University Taichung Taiwan; ^13^ Graduate Institute of Biomedical Sciences China Medical University Taichung Taiwan

**Keywords:** ADAM‐10, betel quid, cigarette, oral squamous cell carcinoma, single‐nucleotide polymorphisms

## Abstract

A disintegrin and metalloproteinase domain‐containing protein 10 (ADAM‐10) involves in the tumour progression, but the impacts of single‐nucleotide polymorphism (SNP) of *ADAM‐10* on oral squamous cell carcinoma (OSCC) remain unclear. The aim of this study was to investigate the influence of SNP of *ADAM‐10* on the clinical features of OSCC in male Taiwanese. Five loci of *ADAM‐10* SNPs including rs653765 (C/T), rs2305421 (A/G), rs514049 (A/C), rs383902 (T/C) and rs2054096 (A/T) were genotyped by TaqMan allelic discrimination in 1138 OSCC patients and 1199 non‐OSCC individuals. The *ADAM‐10* SNP rs2305421 GG (AOR: 1.399, 95% CI: 1.045–1.874, *p* = 0.024) and G allele (AOR: 1.170, 95% CI: 1.012–1.351, *p* = 0.034) illustrated a significantly higher genotypic frequencies in the OSCC group compared to the distribution of the *ADAM‐10* SNP rs2305421 AA wild type. In the subgroup analysis, the *ADAM‐10* SNP rs383902 TC+CC was significantly correlated to tumour size larger than T2 in betel quid chewer (AOR: 1.375, 95% CI: 1.010–1.872, *p* = 0.043), while the *ADAM‐10* SNP rs653765 CT+TT was significantly associated with tumour size larger than T2 in cigarette smoker (AOR: 1.346, 95% CI: 1.023–1.772, *p* = 0.034). The results from The Cancer Genome Atlas revealed highest *ADAM‐10* mRNA level in T2 stage of current smokers with head and neck squamous cell carcinoma (HNSCC). In conclusions, the *ADAM‐10* SNP rs2305421 G allele is associated with the presence of OSCC, and the *ADAM‐10* SNP rs383902 TC+CC and *ADAM‐10* SNP rs653765 CT+TT correlates to large tumour size in specific conditions.

## 1 INTRODUCTION

The oral squamous cell carcinoma (OSCC) is a common cancer which belongs to the group of head and neck squamous carcinoma.^1^ The initial presentation of OSCC may be a plaque in the oral cavity and oral lesion including oral potentially malignant disorders can progress to OSCC.^2^, ^3^, ^4^ The common treatment option for OSCC including the surgical incision and radiotherapy,^1^, ^5^ and the immunotherapy for the OSCC have been developed in recent years.^6^ The overall 5‐year survival rate of OSCC had improved from 59% to 70% in the past decades in Taiwan.^7^

The presence or progression of OSCC is related to certain predisposing factors in previous publications including cigarette smoking, betel quid chewing and alcohol drinking.^1^, ^8^, ^9^ On the other hand, the existence of human papillomavirus (HPV) is a risk factor for OSCC and the HPV 16 species contributes to the majority of HPV‐related OSCC.^10^ About the field of genetics, numerous studies have shown that the single‐nucleotide polymorphism (SNP) may involve in the OSCC development.^11^, ^12^, ^13^, ^14^, ^15^

A disintegrin and metalloproteinase domain‐containing protein 10 (ADAM‐10) belong to a group of transmembrane and secreted proteins and involves in the pathway of cell migration, cell adhesion, proteolysis and cell signalling.^16^ In previous studies, the ADAM‐10 is also associated with certain type of malignancies such as the prostate cancer and pancreatic cancer.^17^, ^18^ Besides, the silencing of ADAM‐10 can influence the invasion, migration and proliferation of specific human tongue squamous cell carcinoma cell line,^19^ and the up‐regulation of ADAM‐10 was observed in patients with OSCC.^20^ Moreover, the SNPs rs514049 and rs514049 of *ADAM‐10* are involved in the hepatocellular carcinoma progression.^21^ Still, whether the genetic polymorphism of *ADAM‐10* will affect the clinical condition of OSCC is unknown. Because the SNP of *ADAM‐10* could influence the clinical course of other disease and ADAM‐10 itself is related to OSCC development,^20^, ^22^ such possibility may exist which need additional elucidation.

In this study, we hypothesized that *ADAM‐10* SNPs (rs653765, rs2305421, rs514049, rs383902 and rs2054096) might influence the susceptibility of OSCC in an Asian male population. The effects of several risk factors of OSCC were also considered in our analytic model.

## 2 MATERIALS AND METHODS

### 2.1 Subject selection

This study was executed in the Chung Shan Medical University Hospital which locates in the central Taiwan region. After obtaining the informed consents, 1138 men with the diagnosis of OSCC participated this study. The diagnosis standard of OSCC in our study is according to the result of incisional biopsy which was judged by an experienced oncologist/otolaryngologist. For the comparison, another 1199 non‐cancer male constituted the control group. We obtained the demographic data such as the age, betel quid chewing, cigarette smoking and alcohol drinking from the medical documents. In addition, the clinical stage, tumour–node–metastasis (TNM) stage and degree of cell differentiation were judged with the assistance of AJCC 7th chart. Our study was approved by the Institutional Review Boards of Chung Shan Medical University Hospital (project identification code: CS1‐21151) and written informed consents were signed by all the patients in our study before the start of our project. For the processing of *ADAM‐10* genes and associated SNPs, we draw venous blood in the study population and the venous blood samples were preserved in ethylenediaminetetraacetic acid‐containing tubes. Immediately after the sample acquisition, those venous blood were centrifuged and placed in a laboratory refrigerator at −80°C.

### 2.2 DNA extraction and analysed *ADAM‐10* SNP with real‐time PCR

Five *ADAM‐10* SNPs like the rs653765 (C/T), rs2305421 (A/G), rs514049 (A/C), rs383902 (T/C) and rs2054096 (A/T) were enrolled in the analysis model since the previous studies illustrated the potential correlation between these *ADAM‐10* SNPs and other carcinoma.^21^ The DNA extraction and analyses procedures in our study were similar to the methods in our previous research.^23^ We separated genome and DNA from leukocytes in venous blood samples via QIAamp DNA kits (Qiagen, Valencia, Valencia, CA, USA) based on the manufacture's instruction of DNA isolation.^24^ Then, these isolated DNA was placed in a refrigerator with internal temperature of −20°C. Then genetic polymorphism of the five *ADAM‐10* SNPs rs653765 (C/T), rs2305421 (A/G), rs514049 (A/C), rs383902 (T/C) and rs2054096 (A/T) were detected via the usage of ABI StepOne Real‐Time PCR System (Applied Biosystems, Foster City, California), and the ADAM‐10 SNPs were further evaluated and optimized by the application of SDS version 3.0 software.

### 2.3 TCGA database survey

The Cancer Genome Atlas (TCGA) is a nationwide program held by National Cancer Institute and National Human Genome Research Institute in the United States. This genome database enrolled more than 20 thousands of participants from 33 cancer types, and the results analysed from blood sample and pathological tissue are available in public. We obtained the ADAM‐10 mRNA levels and clinical characters of head and neck squamous cell carcinoma (HNSCC) from TCGA database, and a subgroup analysis about the correlation between ADAM‐10 mRNA levels and OSCC clinical stage was made in non‐smoker and current smoker.

### 2.4 Statistical analysis

We used the SAS version 9.4 (SAS Institute Inc, NC, USA) for the analyses included in our study. Firstly, the descriptive analyses were used to present the demographic data between the OSCC group and control groups, and Mann–Whitney *U* test or Fisher's exact test was used to evaluate the difference of demography between the two groups. Then, the genotypic frequencies of each *ADAM‐10* SNP was compared between the OSCC group and control group via the application of multiple logistic regression models after controlling for age, betel quid chewing, cigarette smoking and alcohol drinking. The adjusted odds ratio (AOR) and confidence intervals (CI) were produced in this process. We divided the OSCC patients into those with betel quid chewing and those without such habit, and we estimated the AOR and 95% CI between OSCC clinical characters and genotypic frequencies of *ADAM‐10* SNP rs383902 in each subgroup by multiple logistic regression models after controlling for age, alcohol drinking and cigarette smoking. Similarly, the AOR and 95% CI of *ADAM‐10* SNP rs653765 genotypic frequencies for OSCC clinical statuses were estimated by multiple logistic regression models after controlling for age, betel quid chewing and alcohol drinking in cigarette smokers and non‐cigarette smokers of OSCC. A *p* value lower than 0.05 was defined as statistical significance.

## 3 RESULTS

### 3.1 Basic information between the two groups

The demographic data between the two groups are presented in Table 1. The age distribution was similar between the two groups (*p* = 0.670), while the ratio of betel quid chewing, cigarette smoking and alcohol drinking were all significantly higher in the OSCC group than the control group (all *p* < 0.001). The detail of OSCC characters are also presented in Table 1.

**TABLE 1:** The distributions of demographical characteristics in 1199 controls and 1138 male patients with oral squamous cell carcinoma.

Variables	Controls (*N* = 1199)	OSCCs (*N* = 1138)	*p* Value
*Age (years)*			0.670
≦55	610 (50.9%)	589 (51.8%)	
>55	589 (49.1%)	549 (48.2%)	
*Betel quid chewing*			<0.001^a^
No	1000 (83.4%)	296 (26.0%)	
Yes	199 (16.6%)	842 (74.0%)	
*Cigarette smoking*			<0.001^a^
No	563 (47.0%)	171 (15.0%)	
Yes	636 (53.0%)	967 (85.0%)	
*Alcohol drinking*			<0.001^a^
No	962 (80.2%)	603 (53.0%)	
Yes	237 (19.8%)	535 (47.0%)	
*Stage*			
I + II		540 (47.5%)	
III + IV		598 (52.5%)	
*Tumour T status*			
T1 + T2		599 (52.6%)	
T3 + T4		539 (47.4%)	
*Lymph node status*			
N0		757 (66.5%)	
N1 + N2 + N3		381 (33.5%)	
*Metastasis*			
M0		1128 (99.1%)	
M1		10 (0.9%)	
*Cell differentiation*			
Well differentiated		162 (14.2%)	
Moderately or poorly differentiated		976 (85.8%)	

Abbreviations: *N*, number; OSCC, oral squamous cell carcinoma.

^a^ Denotes significant difference between the two groups.

### 3.2 Genotypic frequencies of ADAM‐10 SNPs in OSCC and non‐OSCC male population

The distribution frequencies of the five *ADAM‐10* SNPs between OSCC patients and control group are demonstrated in Table 2. In the control group, the genotypic frequencies of *ADAM‐10* rs653765, rs2305421, rs514049 and rs2054096 were in Hardy–Weinberg equilibrium (*p* > 0.05). The *ADAM‐10* SNP rs2305421 GG revealed a significantly higher genotypic frequencies (AOR: 1.399, 95% CI: 1.045–1.874, *p* = 0.024) in the OSCC group compared to the distribution of the AA wild type (Table 2). The genotypic frequencies of other *ADAM‐10* SNPs between OSCC and control groups did not show significant difference compared to the wild type (all 95% CIs included 1). Also, the *ADAM‐10* SNP rs2305421 G allele owned a higher distribution in the OSCC group (AOR: 1.170, 95% CI: 1.012–1.351, *p* = 0.034) compared to the *ADAM‐10* SNP rs2305421 A allele wild type (Table 2).

**TABLE 2:** Odds ratio and 95% confidence interval of oral cancer associated with ADAM‐10 genotypic frequencies in male population.

Variables	Controls (*N* = 1199) (%)	OSCCs (*N* = 1138) (%)	AOR (95% CI)
*rs653765*			
CC	801 (66.8%)	790 (69.4%)	1.000 (reference)
CT	370 (30.9%)	316 (27.8%)	0.911 (0.729–1.139)
TT	28 (2.3%)	32 (2.8%)	1.011 (0.533–1.916)
CT+TT	398 (33.2%)	348 (30.6%)	0.919 (0.740–1.141)
C allele	1972 (82.2%)	1896 (83.3%)	1.000 (reference)
T allele	426 (17.8%)	380 (16.7%)	0.941 (0.779–1.136)
*rs2305421*			
AA	457 (38.1%)	373 (32.8%)	1.000 (reference)
AG	551 (46.0%)	543 (47.7%)	1.070 (0.855–1.339)
GG	191 (15.9%)	222 (19.5%)	1.399 (1.045–1.874)*
AG+GG	742 (61.9%)	765 (67.2%)	1.152 (0.934–1.421)
A allele	1465 (61.1%)	1289 (56.6%)	1.000 (reference)
G allele	933 (38.9%)	987 (43.4%)	1.170 (1.012–1.351)†
*rs514049*			
AA	1056 (88.1%)	1004 (88.2%)	1.000 (reference)
AC	142 (11.8%)	132 (11.6%)	0.886 (0.647–1.212)
CC	1 (0.1%)	2 (0.2%)	2.437 (0.099–59.701)
AC+CC	143 (11.9%)	134 (11.8%)	0.894 (0.654–1.222)
A allele	2254 (94.0%)	2140 (94.0%)	1.000 (reference)
C allele	144 (6.0%)	136 (6.0%)	0.909 (0.673–1.228)
*rs383902*			
TT	853 (71.1%)	830 (72.9%)	1.000 (reference)
TC	327 (27.3%)	287 (25.2%)	0.894 (0.711–1.125)
CC	19 (1.6%)	21 (1.9%)	0.926 (0.430–1.994)
TC+CC	346 (28.9%)	308 (27.1%)	0.896 (0.716–1.122)
T allele	2033 (84.8%)	1947 (85.5%)	1.000 (reference)
C allele	365 (15.2%)	329 (14.5%)	0.913 (0.748–1.116)
*rs2054096*			
AA	363 (30.2%)	329 (28.9%)	1.000 (reference)
AT	587 (49.0%)	542 (47.6%)	0.913 (0.722–1.156)
TT	249 (20.8%)	267 (23.5%)	1.088 (0.820–1.422)
AT+TT	836 (69.8%)	809 (71.1%)	0.965 (0.774–1.203)
A allele	1313 (54.8%)	1200 (52.7%)	1.000 (reference)
T allele	1085 (45.2%)	1076 (47.3%)	1.035 (0.897–1.193)

Abbreviations: AOR, Adjusted odds ratio; CI, Confidence intervals were estimated by logistic regression models; *N*, number; OR, Odds ratio; OSCC, oral squamous cell carcinoma.

* Denotes significant difference between groups, *p* = 0.024.

^†^ Denotes significant difference between groups, *p* = 0.034.

### 3.3 Subgroup analyses for the correlation between ADAM‐10 SNP genotypic frequencies and clinical conditions of OSCC

In the subgroup analysis, the presence of *ADAM‐10* SNP rs383902 TC+CC was significantly correlated to an OSCC tumour size larger than T2 in betel quid chewer (AOR: 1.375, 95% CI: 1.010–1.872, *p* = 0.043) (Table 3). Moreover, the *ADAM‐10* SNP rs653765 CT+TT was significantly associated with an OSCC tumour size larger than T2 in cigarette smoker (AOR: 1.346, 95% CI: 1.023–1.772, *p* = 0.034; Table 4). The other SNPs of *ADAM‐10* rs383902 and rs653765 did not correlate to clinical characters of OSCC significantly in specific population (all *p* > 0.05) (Tables 3 and 4).

**TABLE 3:** Odds ratio and 95% confidence intervals of clinical statuses associated with genotypic frequencies of ADAM‐10 rs383902 in male oral squamous cell carcinoma patients.

Variables	Total (*N* = 1138)			Betel quid chewer (*N* = 842)			Non‐betel quid chewer (*N* = 296)		
	TT (*N* = 830)	TC + CC (*N* = 308)	*p* Value	TT (*N* = 621)	TC+CC (*N* = 221)	*p* Value	TT (*N* = 209)	TC+CC (*N* = 87)	*p* Value
*Clinical stage*									
Stage I + II	402 (48.4%)	138 (44.8%)	0.325	299 (48.2%)	104 (47.1%)	0.799	103 (49.3%)	34 (39.1%)	0.127
Stage III + IV	428 (51.6%)	170 (55.2%)		322 (51.8%)	117 (52.9%)		106 (50.7%)	53 (60.9%)	
*Tumour size*									
≦T2	450 (54.2%)	149 (48.4%)	0.066	340 (54.8%)	104 (47.1%)	0.043*	110 (52.6%)	45 (51.7%)	0.814
>T2	380 (45.8%)	159 (51.6%)		281 (45.2%)	117 (52.9%)		99 (47.4%)	42 (48.3%)	
*Lymph node metastasis*									
No	550 (66.3%)	207 (67.2%)	0.662	419 (67.5%)	152 (68.8%)	0.672	131 (62.7%)	55 (63.2%)	0.851
Yes	280 (33.7%)	101 (32.8%)		202 (32.5%)	69 (31.2%)		78 (37.3%)	32 (36.8%)	
*Metastasis*									
M0	824 (99.3%)	304 (98.7%)	0.381	617 (99.4%)	218 (98.6%)	0.325	207 (99.0%)	86 (98.9%)	0.841
M1	6 (0.7%)	4 (1.3%)		4 (0.6%)	3 (1.4%)		2 (1.0%)	1 (1.1%)	
*Cell differentiated grade* ^a^									
Well	126 (15.2%)	36 (11.7%)	0.136	99 (15.9%)	27 (12.2%)	0.182	27 (12.9%)	9 (10.3%)	0.483
Moderate or poor	704 (84.8%)	272 (88.3%)		522 (84.1%)	194 (87.8%)		182 (87.1%)	78 (89.7%)	

Abbreviation: *N*, Number.

* Denotes significant difference of clinical statuses between different genotype, adjusted odds ratio with 95% confidence intervals: 1.375 (1.010–1.872).

^a^ Cell differentiate grade: grade I: well differentiated; grade II: moderately differentiated; and grade III: poorly differentiated.

**TABLE 4:** Odds ratio and 95% confidence intervals of clinical statuses associated with genotypic frequencies of ADAM‐10 rs653765 in male oral squamous cell carcinoma patients.

Variables	Total (*N* = 1138)			Cigarette smoker (*N* = 967)			Non‐cigarette smoker (*N* = 171)		
	CC (*N* = 790)	CT+TT (*N* = 348)	*p* Value	CC (*N* = 671)	CT+TT (*N* = 296)	*p* Value	CC (*N* = 119)	CT+TT (*N* = 52)	*p* Value
*Clinical stage*									
Stage I + II	381 (48.2%)	159 (45.7%)	0.444	320 (47.7%)	136 (46.0%)	0.647	117 (46.6%)	30 (44.1%)	0.274
Stage III + IV	409 (51.8%)	189 (54.3%)		351 (52.3%)	160 (54.0%)		134 (53.4%)	38 (55.9%)	
*Tumour size*									
≦T2	429 (54.3%)	170 (48.9%)	0.087	373 (55.6%)	143 (48.3%)	0.034*	120 (47.8%)	33 (48.5%)	0.598
>T2	361 (45.7%)	178 (51.1%)		298 (44.4%)	153 (51.7%)		131 (52.2%)	35 (51.5%)	
*Lymph node metastasis*									
No	521 (66.0%)	236 (67.8%)	0.513	445 (66.3%)	202 (68.2%)	0.517	159 (63.4%)	44 (64.7%)	0.944
Yes	269 (34.0%)	112 (31.2%)		226 (33.7%)	94 (31.8%)		92 (36.6%)	24 (35.3%)	
*Metastasis*									
M0	785 (99.4%)	343 (98.6%)	0.192	667 (99.4%)	291 (98.3%)	0.105	248 (98.8%)	68 (100.0%)	–
M1	5 (0.6%)	5 (1.4%)		4 (0.6%)	5 (1.7%)		3 (1.2%)	0 (0.0%)	
*Cell differentiated grade* ^a^									
Well	120 (15.2%)	42 (12.1%)	0.167	104 (15.5%)	40 (13.5%)	0.440	16 (13.5%)	2 (3.8%)	0.086
Moderate or poor	670 (84.8%)	306 (87.9%)		567 (84.5%)	256 (86.5%)		103 (86.5%)	50 (96.2%)	

Abbreviation: *N*, Number.

* Denotes significant difference of clinical statuses between different genotype, adjusted odds ratio with 95% confidence intervals: 1.346 (1.023–1.772).

^a^ Cell differentiate grade: grade I: well differentiated; grade II: moderately differentiated; and grade III: poorly differentiated.

### 3.4 The relationship between ADAM‐10 mRNA levels and clinical characters of head and neck squamous cell carcinoma in The Cancer Genome Atlas program

**FIGURE 1:**
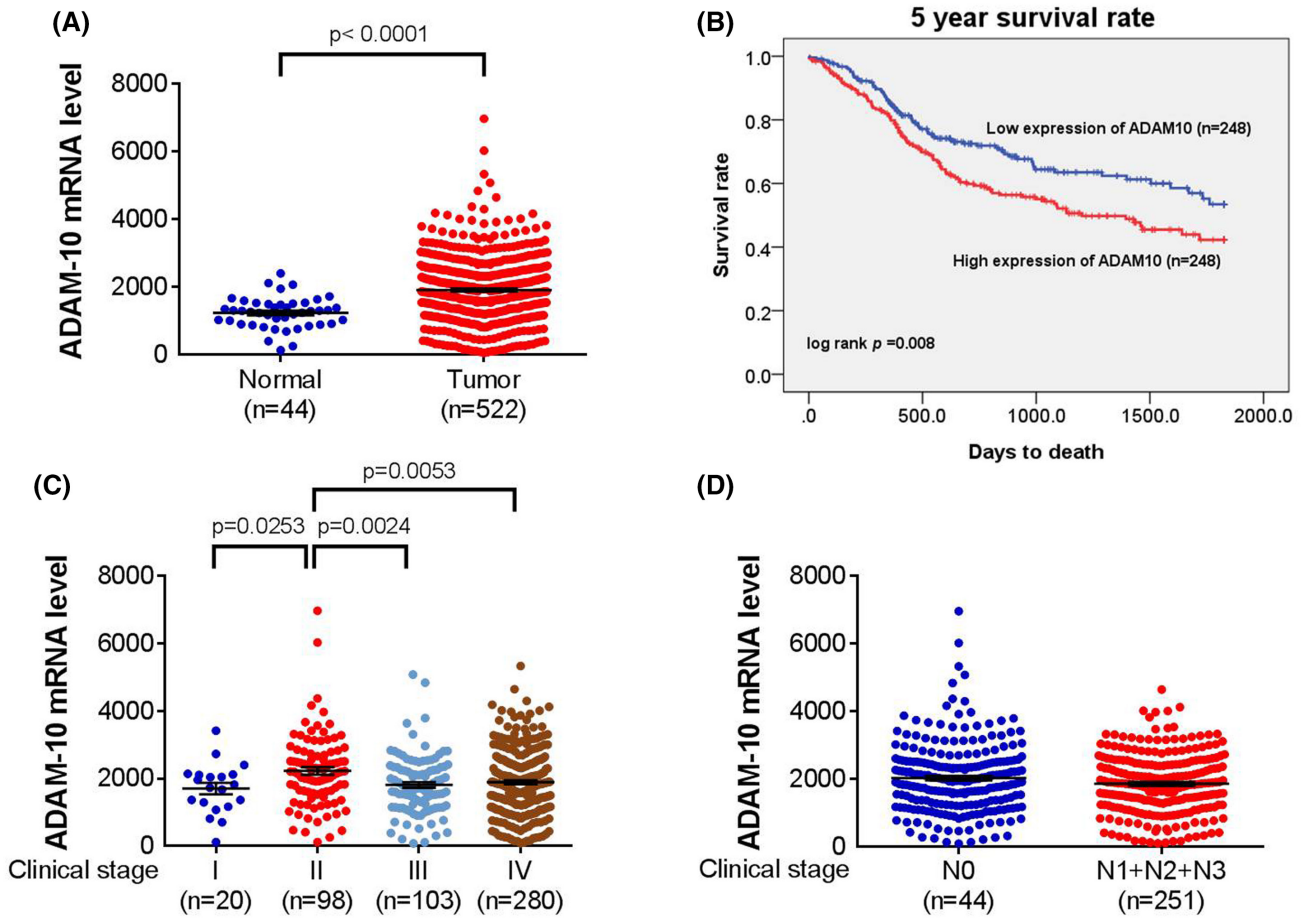
The correlation of ADAM‐10 mRNA level and the clinical features of head and neck squamous cell carcinoma in The Cancer Genome Atlas program. (A) The expression of ADAM‐10 mRNA in head and neck squamous cell carcinoma patients and control group. (B) The 5‐year survival rate between patients with different ADAM‐10 mRNA level. (C) The correlation between ADAM‐10 mRNA level and clinical stage of head and neck squamous cell carcinoma. (D) The correlation between ADAM‐10 mRNA level and N status of head and neck squamous cell carcinoma.

**FIGURE 2:**
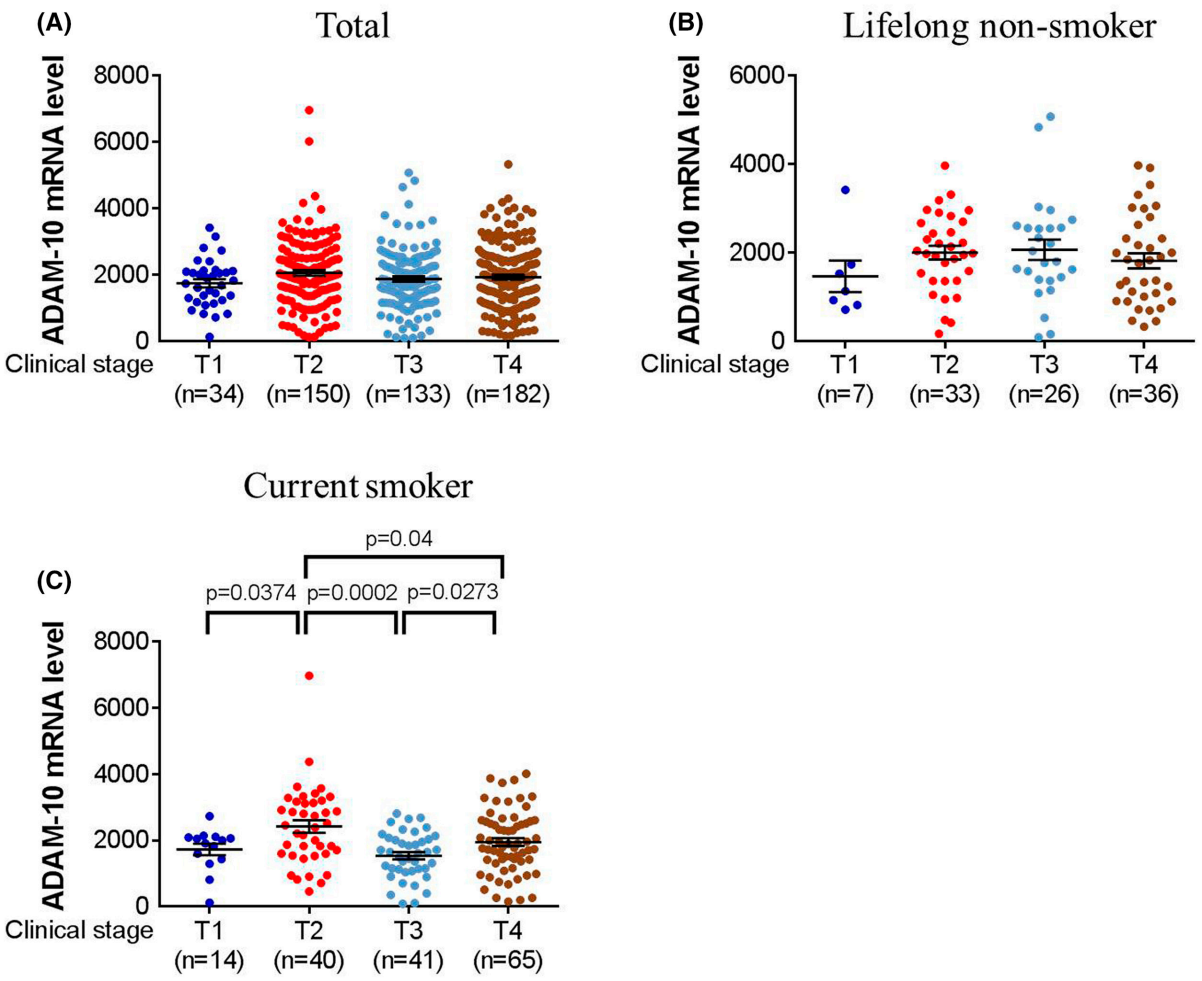
The correlation of ADAM‐10 mRNA level and the tumour size of head and neck squamous cell carcinoma in The Cancer Genome Atlas. (A) The correlation between ADAM‐10 mRNA level and T status of head and neck squamous cell carcinoma. (B) The correlation between ADAM‐10 mRNA level and T status of head and neck squamous cell carcinoma in lifelong non‐smoker. (C) The correlation between ADAM‐10 mRNA level and T status of head and neck squamous cell carcinoma in current smoker.

Concerning the results from TCGA, the ADAM‐10 mRNA level was significantly higher in the HNSCC group than the control group (*p* < 0.0001) (Figure 1A) and associated with lower 5‐year survival rate of HNSCC (*p* = 0.008) (Figure 1B). The ADAM‐10 mRNA level was significantly higher in the clinical stage II of HNSCC compared to other clinical stages (all *p* < 0.05) (Figure 1C) but did not associate with the N statuses (Figure 1D). About the T statuses of HNSCC group, the ADAM‐10 mRNA expression was not correlated to the T status in all HNSCC group (Figure 2A) and lifelong non‐smoker (Figure 2B), while the ADAM‐10 mRNA expression was significantly higher the T2 status than other T statuses in current smoker (all *p* < 0.05) (Figure 2C).

## 4 DISCUSSION

Briefly, our study demonstrated the higher distribution frequency of *ADAM‐10* SNP rs2305421 GG variant and G allele in the individuals with OSCC than the control group. Furthermore, the *ADAM‐10* SNP rs383902 TC+CC variant showed a strong correlation to higher OSCC T status in betel quid chewer. On the other hand, the *ADAM‐10* SNP rs653765 CT+TT variant related to the larger OSCC T status in the cigarette smoker which may correspond to the findings of HNSCC and ADAM‐10 mRNA level in the TCGA.

The ADAM‐10 and its genetic polymorphism had been proven to influence various diseases including neoplasms according to previous literatures.^25^, ^26^, ^27^ The main function of ADAM‐10 is the cell reaction like the cell migration and cellular signal pathways.^16^ In a previous study, the ADAM‐10 can alter the CD44 function at different levels and especially in the malignancies which with high CD44 level.^28^ Also, the ADAM‐10 can regulate the E‐cadherin pathway which controls the cell adhesion, cell differentiation and tissue development.^29^ On the other hand, the ADAM‐10 is associated with the disease course of some diseases. The Alzheimer's disease may be retarded by ADAM‐10 which can reduce the generation of amyloid‐β peptides,^26^ and the *ADAM‐10* SNP rs2305421 is related to late‐onset Alzheimer's disease.^22^ In another study, the mutation of ADAM‐10 which causes loss‐of‐function can be a predisposing factor for the development of Alzheimer disease.^30^ In addition, the *ADAM‐10* SNP rs653765 was correlated to the formation of atherosclerotic cerebral infarction, thus may own therapeutic implications.^31^ About the relationship between ADAM‐10 and malignancy, the higher expression of ADAM‐10 may trigger the metastasis of prostate cancer via the ephrin‐A5 shedding.^17^ And the patients carry *ADAM‐10* SNPs rs514049 and rs514049 have a higher chance to develop hepatocellular carcinoma in more advanced TNM stage.^21^ On the other side, the clinical manifestations of OSCC are influenced by several genetic factors.^32^ For example, the ADAM‐10 expression is significantly correlated to the presence of OSCC.^20^ Besides, the fibroblast growth factor receptor 4 with Arg allele carrier polymorphism can attribute to higher chance of nodal metastasis patients with OSCC than the wild type.^33^ Because the *ADAM‐10* SNP can affect the clinical course of some cancers and the OSCC owns correlation to the ADAM‐10 expression.^20^, ^25^ Furthermore, previous study showed that patients who carried the *ADAM‐10* SNP rs653765 in the promoter region were presented with higher ADAM‐10 mRNA levels in a Chinese population.^31^ Thus, we speculate that the SNP of *ADAM‐10* may alter the clinical condition of OSCC. This hypothesis was partially supported by the findings of our study and the TCGA database.

In this study, the *ADAM‐10* SNP rs2305421 GG variant and G allele demonstrated a higher distribution in the individuals with OSCC than the individuals without OSCC. To the best of our knowledge, few study survey the relationship between the genotypic frequencies of *ADAM‐10* and the presence of OSCC. Moreover, the multiple logistic regression of our study considered the effects of several known risk factors of OSCC which including the age, betel quid chewing, cigarette smoking and alcohol drinking. Consequently, the presence of *ADAM‐10* SNP rs2305421 GG and G allele may independently correlate to the development of OSCC in the Taiwanese male population. About the effect of this *ADAM‐10* SNP in other disease, a previous study revealed the correlation between *ADAM‐10* SNP rs2305421 G variant and late‐onset Alzheimer's disease,^22^ and another study also found the possible relationship between the *ADAM‐10* SNP rs2305421 G and Alzheimer's disease.^34^ Thus, the *ADAM‐10* SNP rs2305421 G allele may be a risk factor for many diseases. According to the data from the TCGA, the mRNA expression of ADAM‐10 was significantly higher in the HNSCC group than that in the non‐HNSCC individuals. In general, the OSCC is a subtype of HNSCC and both cancers share similar predisposing factors and histological features.^35^ Consequently, we assumed that the *ADAM‐10* SNP rs2305421 G allele may cause a higher expression of ADAM‐10 mRNA and increase the possibility of OSCC. Still, further experiment is warranted to prove this concept.

In the subgroup analyses, the *ADAM‐10* SNP rs383902 TC+CC and *ADAM‐10* SNP rs653765 CT+TT variants are associated with higher OSCC T status in the betel quid chewer and cigarette smoker respectively. Similar to the multiple logistic regression for the comparison between OSCC and control groups, several predisposing factors of OSCC were adjusted in the two subgroup analyses. The two *ADAM‐10* SNPs may be independent predictor for higher T status in the two subgroups. The betel quid chewing can also affect the clinical course of oral cancer under the expression of dipeptidyl peptidase IV SNP rs2268889,^36^ and the *ADAM‐10* also owns the cell migration ability that is similar to dipeptidyl peptidase IV, thus the SNP of *ADAM‐10* may show similar effect.^16^, ^37^ The cigarette smoker is vulnerable to the atherosclerotic cerebral infarction and the *ADAM‐10* SNP rs653765 also showed certain effect on the atherosclerotic cerebral infarction.^31^ Maybe, the cigarette components and the *ADAM‐10* variant caused by *ADAM‐10* SNP rs653765 have synergic effect on various disease developments. Moreover, previous studies also showed that ADAMs was involved in betel quid or tobacco‐related carcinogenesis.^38^, ^39^ Concerning the results of TCGA, the ADAM‐10 mRNA level was highest in the T2 stage of HNSCC in current smoker. Combing the findings from our study and the TCGA database, it may be possible that the *ADAM‐10* SNP rs653765 CT+TT variant trigger a larger tumour size of OSCC in smoker, but the expression of ADAM‐10 mRNA was down‐regulated by the presence of this genetic polymorphism and the cigarette smoking. Since the higher expression of ADAM‐10 mRNA was correlated to lower 5‐year survival rate of HNSCC, the role of *ADAM‐10* SNP rs653765 CT+TT variant in the prognosis of OSCC and HNSCC need additional evaluation.

In conclusion, the *ADAM‐10* SNP rs2305421 G allele is higher in the OSCC patients compared to non‐OSCC group. Furthermore, the *ADAM‐10* SNP rs383902 and *ADAM‐10* SNP rs653765 variants are correlated to larger tumour size of OSCC in specific population. Consequently, a survey of *ADAM‐10* genetic variant might be recommended for OSCC men especially for patients with betel quid chewing and cigarette smoking. Further large‐scale prospective study to evaluate whether the genetic polymorphism of ADAM‐10 would affect the treatment outcome of OSCC in male is mandatory.

## AUTHOR CONTRIBUTIONS

**Yi‐Tzu Chen:** Conceptualization (equal); writing – original draft (equal); writing – review and editing (equal). **Chiao‐Wen Lin:** Writing – original draft (equal). **Ying‐Erh Chou:** Methodology (equal). **Shih‐Chi Su:** Methodology (equal). **Lun‐Ching Chang:** Methodology (equal). **Chia‐Yi Lee:** Writing – original draft (equal). **Ming‐Ju Hsieh:** Conceptualization (equal); writing – original draft (equal); writing – review and editing (equal). **Shun‐Fa Yang:** Conceptualization (equal); writing – original draft (equal); writing – review and editing (equal).

## FUNDING INFORMATION

This study was supported by Chung Shan Medical University Hospital, Taiwan (CSH‐2021‐C‐016).

## CONFLICT OF INTEREST STATEMENT

The authors declare that there is no conflict of interest.

## Data Availability

The data used to support the findings of this study are available from the corresponding author upon request.
